# Sleep characteristics in child and adolescent offspring of parents with bipolar disorder: a case control study

**DOI:** 10.1186/s12888-017-1361-8

**Published:** 2017-05-26

**Authors:** Antonin Sebela, Tomas Novak, David Kemlink, Michal Goetz

**Affiliations:** 1 National Institute of Mental Health, Topolova 748, 250 67 Klecany, Czech Republic; 2 First Faculty of Medicine, Charles University in Prague, Katerinska 32, 121 08 Prague 2, Czech Republic; 3 Second Faculty of Medicine, Charles University in Prague, V Uvalu 84, 5, 15006 Prague, Czech Republic; 4 Third Faculty of Medicine, Charles University in Prague, Ruska 87, 10, 100 00 Prague, Czech Republic; 5 Department of Neurology, General Teaching Hospital and First Faculty of Medicine, Charles University in Prague, Katerinska 30, 128 21 Prague 2, Czech Republic; 6 Department of Child and Adolescent Psychiatry, Motol University Hospital, V Uvalu 84, 5, 15006 Prague, Czech Republic

**Keywords:** Actigraphy, Offspring, Bipolar disorder, Sleep, High-risk, Child, Adolescent

## Abstract

**Background:**

Impairment of sleep and circadian rhythm is a typical feature of bipolar disorder (BD). We carried out an exploratory cross-sectional case-control study to extend the knowledge of sleep characteristics in offspring at risk for BD.

**Methods:**

We investigated 42 offspring of bipolar parents (OB) (mean age 12.5 ± 3.2) and 42 sex and age matched comparison offspring of healthy parents. We administered the Pediatric Sleep Questionnaire, the Morningness/Eveningness Questionnaire and The General Behavior Inventory Sleep Subscale (GBISS) to assess circadian preference, and to identify sleep impairment symptoms. In addition, the participants completed 14 days of actigraphy to characterise sleep and wake patterns. The current psychopathology profile was assessed using Kiddie Schedule for Affective Disorders and Schizophrenia.

**Results:**

Prevalence of sleep disturbance symptoms was higher among OB than controls (headache after waking up, 17.9% vs. 2.4%, *p* = 0.03; excessive daytime sleepiness, 38.5% vs. 10.0%, *p* = 0.004; apparent tiredness at wake-up times, 43.6% vs. 15.0%, *p* = 0.007 and nightmares, 21.6% vs. 2.4%, *p* = 0.01), but the differences between groups were not significant after adjusting for current psychopathology. OB had higher GBISS total score (parental version, *p* < 0.001; self-assessment, *p* = 0.07) than the controls. OB had higher preference for eveningness than the controls (*p* = 0.047). According to the actigraphy, OB had longer sleep onset latency (*p* = 0.048) than the controls.

**Conclusion:**

Evidence suggests that the offspring of bipolar parents experience sleep disturbance symptoms, which was associated with psychopathology in this study. Prospective longitudinal sleep studies would clarify whether sleep disturbance could be a predictor of mood disorder onset in this high-risk population.

## Background

Bipolar disorder (BD) is a lifelong psychiatric illness that severely impairs quality of life with an estimated life-time prevalence from 1 to 2% [1]. The usual age of onset for BD is from late adolescence to early adulthood, and high-risk studies have provided evidence of childhood antecedents [2]. Retrospective studies have shown that approximately 30–60% of adults with BD reported an illness onset before 20 years of age [3, 4] and up to one quarter of them recalled that their first mood episode occurred before 13 years of age [5]. However, retrospectively obtained age of onset is both vulnerable to recall bias and focused only on major mood episodes.

Differences in sleep architecture and circadian rhythms are commonly found in adults with BD during manic, depressive and even in euthymic phases of their illness [6, 7]. Sleep disturbance is also a common prodrome of both mania and depression [8] among patients with bipolar. Recently, Cretu et al. [9] found strong associations between poor sleep quality and shorter time to mood episode recurrence in recovered patients with BD.

The clock-like circadian system and sleep homeostatic self-modulation are regulating processes of the sleep-wake cycle [10]. Circadian rhythm disruption is a core feature of BD [11]. Moreover, a recent meta-analysis [12] showed that patients with BD have less stable and more variable circadian activity patterns, even in euthymia, than healthy controls. The period of circadian rhythms is variable among individuals and this variability is marked as chronotype or circadian preference. There are three basic types of chronotype: morning, evening and intermediate [13]. Adult patients with BD mostly have the evening chronotype and this feature is relatively stable [14, 15].

Impaired sleep is also an important aspect of paediatric BD. Individuals with BD may manifest increased sleep onset latency [16] and poorer sleep efficiency [17] than healthy controls. Furthermore, sleep disturbance was found to be an initial symptom of BD in 45% of children with full-blown paediatric BD in a retrospective study by Faedda [18]. Proper sleep is also important for all children, as strong evidence suggests that impaired sleep in children and adolescents negatively impacts their cognitive and behavioural functioning [19].

BD is highly heritable, with up to 85% of variance in risk determined by genetic factors [20] and family history of BD is the strongest predictor for the development of this illness [21]. Systematic prospective assessment of the offspring of bipolar parents (OB) is therefore the approach of choice to obtain information on the early stages and the development of BD [22]. Evidence from prospective studies of OB suggests that sleep disturbances are prodromal symptoms of BD [23, 24]. Despite sleep disorders and disturbances being mentioned as initial stages in the developmental model of BD [25], information about sleep characteristics of OB is relatively sparse.

Sleep questionnaires and sleep diaries are common subjective methods for evaluating sleep habits and chronotype. Actigraphy and polysomnography are useful objective methods for evaluating sleep characteristics in patients with BD [26]. Each of these methods has its individual advantages and disadvantages. Actigraphic assessment of sleep parameters has been validated in different studies using polysomnography [27], although it has limitations, which should be mentioned. In comparison to polysomnography, actigraphy has a low ability to detect wakefulness during sleep [28]. Further, it does not mirror the polysomnography in estimating sleep onset latency [29].

Polysomnography records multiple electrophysiological parameters at one time and thus can detect many types of sleep disturbances; it is the gold standard for objective assessment of macro-structural sleep parameters and sleep disorders. However, the data are obtained in a sleep laboratory, therefore, the number of nights that can be recorded for each individual subject is limited and thus insufficient for assessment of sleep-wake cycle stability. The wrist worn actigraphy has the advantage of being able to evaluate sleep patterns at home, and can be used for longer periods of time. On the other hand, actigraphy measures movement and therefore it is a proxy measure of sleep. To the best of our knowledge, only two studies from the large number of OB studies have specifically evaluated sleep characteristics of offspring with genetic and environmental risk of BD due to illness of parent [30, 31].

The first study dedicated to sleep among OB was conducted by Jones et al. [30]. The authors used both subjective and objective methods for assessment of sleep patterns among OB. Seven days of actigraphic measurement were used as an objective method. The only statistical difference in sleep between the OB and the control groups in this study was shorter sleep latency in OB. Furthermore, Levenson et al. [31] used subjective methods for evaluating sleep characteristics of OB. In the OB group, the authors found higher rates of inadequate sleep, poor sleepers, frequent awakenings and circadian evening preference. But after adjusting for lifetime psychopathology, only parental perception of inadequate sleep of the child remained.

Studying sleep disturbances, circadian patterns and preferences in the offspring at risk for BD may pave the way for the development of better methods for early diagnostics, early interventions and potentially also for the prevention of BD. To extend the knowledge of sleep characteristics among offspring at risk for BD, we carried out an exploratory cross-sectional case-control study and applied both subjective and objective methods.

## Methods

### Participants

Recruitment of BD families was done using the Czech Bipolar Disorder Case Registry of the National Institute of Mental Health in Klecany, Czech Republic. This register is a database of patients with BD confirmed by the semi-structured psychiatric interview (Schedule for Affective Disorders and Schizophrenia Lifetime version, SADS-L) [32]. We also asked other psychiatric outpatient services across the Czech Republic to inform adults with BD I or II about our survey. We performed preliminary screening interviews with the families that agreed to participate in the project.

The inclusion criteria were as follows: for parent 1) diagnosis of BD-I or BD-II confirmed by SADS-L, 2) no history of psychotic disorder, 3) written, informed consent with study protocol; for offspring 1) age range 6–17; this age range was chosen because we could detect early symptoms of impairment in various domains of development, 2) no history of severe health complications in the mother during pregnancy capable of potential impairment of intrauterine development, 3) no history of any chronic physical condition and 4) assent with study protocol.

Families were excluded from our study if they had more than one parent with a mood disorder or if they could not comply with the research protocol. All parents completed the standardised SADS-L post-screening research interviews to confirm the diagnosis of BD.

Community control offspring were recruited through advertisements in schools. Exclusion criteria for the control families included the presence of psychiatric disease in the parents, severe health complications during pregnancy capable of potential impairment of intrauterine development of the offspring, and history of chronic physical condition of offspring. We used preliminary interviews to select families to be included in the control sample. We selected families with offspring that matched the sex and age of the offspring from the BD families.

### Offspring assessment

The semi-structural psychiatric interview Kiddie Schedule for Affective Disorders and Schizophrenia Present and Lifetime version (KSADS-PL) [33] was performed independently on both the offspring and the parents by a trained child psychiatrist. The interviewer was not blind to the status of the parent (control vs. bipolar). DSM-IV diagnoses were confirmed based on consensus review by a board-certified child psychiatrist.

#### Sleep questionnaires

We used three questionnaires to assess sleep patterns and disturbances: a) Czech version of the Morningness/Eveningness Questionnaire (MEQ) to assess circadian preferences [34]. The MEQ is a set of ten questions administered to the child; individual answers have a set value. The morningness/eveningness score was derived from responses to questions about preferred timing of activities such as school tests, bedtime and rising time. The morningness/eveningness score range from 10 to 42, with higher scores indicating a greater morning tendency. This scale is validated in children of different age groups [35]. b) The Czech version of the Pediatric Sleep Questionnaire (PSQ) was originally developed for investigation of childhood sleep related breathing disorders and prominent symptom complexes, including snoring (4 items), breathing problems during sleep (4 items), and daytime sleepiness (4 items). Further, the inattentive/hyperactive behaviour scale (6 items) and other items assessing symptoms of impaired sleep are included in the questionnaire. PSQ is completed by the parent concerning his or her offspring using the yes/no/don’t know response format [36]. c) The Czech version of the General Behavior Inventory Sleep Subscale [37] (GBISS) was used to assess sleep disturbances typical for BD. Both parental and self-assessment versions were used. This subscale comprises seven Likert-type items of the General Behavior Inventory [38] that cover symptoms of initial, middle and terminal insomnia. The GBISS scores range from 0 to 21, with higher scores indicating more severe disruption of sleep.

#### Actigraphy

The objective assessment of sleep and wake patterns was analysed by a wrist worn actigraphic device with a tri-axial accelerometer (MotionWatch8, CamNTech, Cambridge, UK, www.camntech.com). The subjects wore the device continuously for two weeks on the non-dominant upper extremity. Offspring recorded if and for how long they removed the actigraphic device, with instruction to take it off only when it could get wet. Parents were instructed to fill the sleep diaries including information about wake up and bed times of their offspring. We also used ordinary wake up and bed times from the PSQ to determine the sleep time. We calculated all variables dependent on the precise record of going to bed/waking up using sleep diaries and the most common time of going to bed/waking up as mentioned by parents in the PSQ. Data were sampled in 60-s epochs, stored digitally and analysed using the MotionWare Software v1.1.20 (CamNTech, Cambridge, UK, www.camntech.com). Both standard sleep macrostructure parameters and non-parametric statistics based on a multiple day recording (Non-Parametric Circadian Rhythm Analysis - NPCRA), as implemented in the system, were used for analyses. For the NPCRA, we selected the following variables: Intra-daily variability (IV), which reflects rhythm fragmentation and has a range of 0 to 2 with higher values indicating higher fragmentation. Inter-daily stability (IS) indicates invariability of the daily rhythm between days in the assessed period and has a range of 0 to 1, where a value of 0 indicates a total lack of rhythm and a value of one indicates a perfectly stable rhythm. Relative amplitude (RA) refers to the quantity of daytime activity. This variable has a range of 0 to 1 with higher values indicating a rhythm with higher amplitude [39]. Actigraphic data analysis was performed by a statistician blind to the status of the offspring (control vs. high-risk).

### Statistical analyses

The demographic and clinical characteristics between the high-risk and control groups were compared using the unpaired t-test, Mann-Whitney U-test, Fisher’s exact tests, and Pearson chi-square test as appropriate.

As most sleep related continuous parameters (GBISS, MEQ, actigraphic measures) were non-normally distributed (assessed by Shapiro-Wilk’s test), the Mann-Whitney U-test was used for comparisons between groups. Dichotomous variables (PSQ) were compared using Fisher’s exact test. Significant differences between groups were consequently adjusted for confounding factors (current psychopathology, psychotropic medication and substance use) using multivariate linear, ordinal or binomial regression analyses to ensure that the differences are attributable to group membership. All statistical procedures were conducted using the STATISTICA software package, version 12.0 (StatSoft, Inc. 2013).

## Results

### Demographic variables and the profile of current psychopathology

We enrolled 34 families with a BD parent (25 cases of BD type I; 9 cases of BD type II) and 33 control families (Table 1). The offspring sample consisted of 42 high-risk offspring and 42 age and sex matched controls (Table 2).

**Table 1:** Parental characteristics

	Bipolar families (*N* = 34)	Control families (*N* = 33)	*p*- value
Families			
Intact family, N, (%)	16 (47.1)	25 (75.8)	0.02^a^
Father’s education, E/H/U, N	3/18/13	0/13/20	0.07^b^
Mather’s education, E/H/U, N	2/24/8	0/11/22	0.001^b^
Probands			
Female/Male	19/15	18/15	1.00^a^
Mood disorders, N, (%)			
BD I	25 (73.5)	n/a	n/a
BD II	9 (26.5)	n/a	n/a
Age at onset of BD, mean ± SD	26.3 ± 10.7	n/a	n/a
No. of BD episodes, mean ± SD	5.4 (2.6)	n/a	n/a
Suicidal attempt, N, (%)	13 (38.2)	0	<0.001^a^
Psychotic symptoms, N, (%)	7 (20.6)	0	0.01 ^a^
Non-mood disorders, N, (%)			
Any anxiety disorder	4 (11.8)	1 (3.0)	0.36^a^
Substance abuse^c^	7 (20.6)	1 (3.0)	0.05^a^

*BD* bipolar disorder, *E/H/U* Elementary/High school/University, *N* number, *n/a* non-applicable, *SD* standard deviation

^a^Fisher’s exact test, ^b^Pearson chi-square test; ^c^includes alcohol marijuana and amphetamines

**Table 2:** Demographic and clinical variables of offspring

	OB	CO	*p*-value
	(*N* = 42)	(*N* = 42)	
Demographic			
Female/Male	17/25	17/25	1.00^a^
Age, mean (SD)	12.5 (3.2)	12.4 (3.1)	0.7^b^
Psychotropic medication^c^, N, (%)	7 (16.7)	0 (0.0)	0.01^a^
Current psychopathology, N, %			
Any BD spectrum disorder^d^	5 (11.9)	0 (0.0)	0.06^a^
Any depressive spectrum disorder^e^	6 (14.3)	0 (0.0)	0.03^a^
Any anxiety disorder^f^	13 (30.9)	4 (9.5)	0.02^a^
Substance use disorder^g^	6 (14.3)	0 (0.0)	0.03^a^
ADHD	10 (23.8)	2 (4.8)	0.03^a^
Not currently mentally ill	17 (40.5)	36 (85.7)	0.001^a^

*ADHD* attention-deficit hyperactivity disorder, *BD* bipolar disorder, *CO* control offspring, *N* number, *NOS* not otherwise specified, *OB* high-risk offspring, *SD* standard deviation

^a^Fisher’s exact test, ^b^unpaired t-test

^c^including antidepressants, psychostimulants and antipsychotics

^d^including BD II, BD NOS and cyclothymia

^e^including major mood disorder, depression NOS, depressive adjustment disorder and dysthymia

^f^including generalised anxiety disorder, social and separation anxiety

^g^including alcohol, marijuana and amphetamines

Five cases of bipolar spectrum disorders were found in the OB group (1 case with BD-II, 3 cases with BD-NOS and 1 case with cyclothymia). No cases of bipolar spectrum disorders were found in the control group. Six cases of depressive spectrum disorders were found in the OB group. No cases of depressive spectrum disorders were found in the control group (Table 2).

### Sleep and circadian variables

For the PSQ, we did not find any significant differences between offspring groups on the main scales (breathing problems during sleep, snoring, daytime sleepiness, inattentive/hyperactive behaviour). We conducted a further analysis on frequency of impaired sleep symptoms (Table 3). Headache after waking up was reported in 17.9% of the OB and 2.4% of controls (*p* = 0.03), excessive daytime sleepiness in 38.5% of the OB and 10.0% of controls (*p* = 0.004), apparent tiredness at wake-up times in 43.6% of the OB and 15.0% of controls (*p* = 0.007), and nightmares in 21.6% of the OB and 2.4% of controls (*p* = 0.01). However, after adjusting for potential confounding factors (current psychopathology, medication and substance use), the significant differences between groups on sleep variables were no longer significant: headache after wake-up (adjusted OR = 2.0; 95% CI: 0.15–28.12; *p* = 0.59), excessive daytime sleepiness (OR = 2.1; 95%CI: 0.49–8.88; *p* = 0.32), apparent tiredness at wake-up times (OR = 1.2; 95% CI: 0.30–4.88; *p* = 0.80), and nightmares (OR = 1.2; 95% CI: 0.07–33.5; *p* = 0.90). Instead, current psychopathology was associated with sleep disturbances as follows: headache after wake-up (ADHD, OR = 17.5, 95% CI: 1.4–220.4, *p* = 0.03 and anxiety disorders, OR = 28.8, 95% CI: 2.2–378.2, *p* = 0.01); excessive daytime sleepiness (ADHD, OR = 6.3, 95% CI: 1.3–31.6, *p* = 0.02 and depressive disorders, OR = 20.4, 95% CI: 1.8–230.8, *p* = 0.01); apparent tiredness at wake-up times (ADHD, OR = 5.8, 95% CI: 1.1–31.6, *p* = 0.04; anxiety, OR = 9.1, 95% CI: 2.0–41.4, *p* = 0.001 and depressive disorders, OR = 17.2, 95%CI: 1.3–234.2, *p* = 0.03); nightmares (substance use, OR = 35.9, 95% CI: 1.4–961.3, *p* = 0.03).

**Table 3:** Parental and self-report of offspring sleep characteristics

	OB	CO	*p*-value
	(*N* = 42)	(*N* = 42)	
PSQ, mean (SD)			
Breathing problems during sleep	0.2 (0.4)	0.2 (0.6)	0.6^a^
Snoring	0.1 (0.2)	0.2 (0.6)	0.1^a^
Daytime sleepiness	0.8 (1.1)	0.5 (0.9)	0.2^a^
Inattentive/hyperactivity behaviour	1.5 (1.5)	0.9 (1.3)	0.05^a^
PSQ, specific items, yes, %			
Visible daytime sleepiness	38.5	10.0	0.004^b,c^
Feels tired after sleep	43.6	15.0	0.007^b,c^
Headaches after wake-up	17.9	2.4	0.03^b,c^
Nightmares	21.6	2.4	0.01^b,c^
GBISS			
Parental version, Total score, mean (SD)	2.3 (3.1)	0.4 (0.9)	0.001^a^
Self–assessment, Total score, mean (SD)	4.6 (4.4)	2.5 (2.3)	0.07^a^
Circadian phase preference			
MEQ, mean (SD)	27.0 (6.4)	30.0 (4.2)	0.047^a^

*CO* control offspring, *GBISS* General Behavior Inventory Sleep Subscale, *MEQ* Morningness-Eveningness Questionnaire, *OB* high-risk offspring, *PSQ* Pediatric Sleep Questionnaire, *SD* standard deviation

^a^Mann–Whitney test;

^b^Fisher’s exact test

^c^non-significant after adjustment for current psychopathology, medication, and substance use (ordinal and binomial logistic regression)

On the GBISS, parents of the OB reported higher total scores compared to parents of control offspring (2.3 ± 3.1 vs. 0.4 ± 0.9; *p* < 0.001; adjusted OR = 1.18; 95%CI: 1.03–1.46; *p* = 0.03). Furthermore, in the parental report of the GBISS scale, OB scored significantly higher on the following items: Increased fatigue and increased sleep, regardless of mood and energy levels (*p* = 0.046), increased fatigue and decreased productivity (*p* = 0.04), middle insomnia regardless of mood and energy levels (*p* = 0.02), and depressed mood with sleep-onset insomnia (*p* = 0.04). There was no group difference on items regarding decreased need for sleep, elation and increased energy with initial insomnia and terminal insomnia. No significant difference in the total score on the GBISS was found in the child and adolescent self-assessment (*p* = 0.07). However, in the analysis of separated items, the cases scored significantly higher on the item concerning depressed mood with sleep-onset insomnia (*p* = 0.002).

In the MEQ, the observed scores were as follows: OB (mean = 27.0; SD = 6.4 and range 13–42), controls (mean = 30.0; SD = 4.22 and range 22–39). This difference was statistically significant (*p* = 0.047). (Table 3).

#### Actigraphy

Thirty-one OB (completion rate 73.8%) and 27 control offspring (completion rate 64.3%) completed the actigraphic part of the study. We excluded data from those offspring exhibiting administration problems (disagreement with actigraphic assessment: 7 OB and 11 controls, long periods of time without wearing actigraphic device: 3 OB and 4 controls, and vitiation of the actigraph during the assessment: 1 OB and 0 controls). The sleep diary completion rate was 29% in the OB group (9 complete sleep diaries) and 66% in the control group (18 complete sleep diaries). When comparing actigraphic results, we found longer sleep latency among the OB compared to the controls, which remained significant even after adjusting for confounding factors (*p* = 0.048). No other intergroup differences on either sleep analysis results nor on the NPCRA were found. Weekdays and weekends were analysed separately for both types of results (Table 4).

**Table 4:** Results of actigraphic measures in offspring

	OB		CO		*p*-value
	(*N* = 31)		(*N* = 27)		
Female/Male	13/18		12/15		1.0^a^
Age, mean, (SD)	12.8 (3.0)		11.9 (3.3)		0.3^b^
Any bipolar spectrum disorder^c^, N (%)	4 (12.9)		0 (0.0)		0.1^a^
Any mood spectrum disorder^d^, N, (%)	3 (9.7)		0 (0.0)		0.2^a^
Any anxiety spectrum disorder^e^, N, (%)	6 (19.4)		4 (14.8)		0.7^a^
ADHD	9 (29.0)		1 (3.7)		0.01^a^
Not currently mentally ill, N, (%)	16 (52)		22 (81)		0.03^a^
Non-Parametric Circadian Rhythm Analysis					
Weekdays	Mean	SD	Mean	SD	
Interdaily Stability	0.70	0.11	0.70	0.10	0.8^c^
Intra-Daily Variability	0.86	0.20	0.81	0.18	0.4^c^
Relative Amplitude	0.95	0.03	0.96	0.02	0.5^c^
Weekends					
Interdaily Stability	0.74	0.10	0.74	0.06	0.4^c^
Intra-Daily Variability	0.75	0.22	0.78	0.17	0.6^c^
Relative Amplitude	0.93	0.09	0.96	0.02	0.4^c^
Sleep macrostructure					
Weekdays	Mean	SD	Mean	SD	
Sleep onset latency, h:min	0:51	0:39	0:39	0:21	0.05^c^
Actual sleep time, h:min	7:35	0:47	7:33	0:50	0.5^c^
Sleep efficiency, %	72.5	5.4	73.6	5.4	0.3^c^
Fragmentation Index	26.6	5.4	29.0	7.1	0.2^c^
Weekends					
Sleep onset latency, h:min	0:56	0:37	0:39	0:21	0.2^c^
Actual sleep time, h:min	7:59	0:47	7:57	0:50	0.8^c^
Sleep efficiency, %	72.3	6.3	72.9	7.0	0.5^c^
Fragmentation Index	30.3	7.6	31.6	8.3	0.5^c^

*ADHD* attention-deficit hyperactivity disorder, *CO* control offspring, *h* hour, *min* minutes, *N* number, *OB* high-risk offspring, *SD* standard deviation

^a^Fisher’s exact test;

^b^unpaired t-test;

^c^Mann-Whitney test

^d^including BD II, BD NOS and cyclothymia

^e^including major mood disorder, depression NOS, depressive adjustment disorder and dysthymia

^f^including generalised anxiety disorder, social and separation anxiety

## Discussion

This cross-sectional exploratory study evaluated sleep characteristics among offspring of bipolar parent using both subjective and objective methods. Our survey is, to our knowledge, only the second, to evaluate sleep characteristics among the offspring of bipolar parent using such a comprehensive approach. With a total sample size from 58 (objective measurements) to 84 (subjective), we should have been able to detect standardised group difference of even small to medium sizes (Cohen’s d of 0.42–0.50) for both the quantitative and qualitative variables if present.

Parents of high-risk offspring reported sleep disturbances in their children more often than the parents of controls. These included: nightmares, feeling of tiredness after sleep, headaches after waking up, excessive daytime sleepiness, increased fatigue, and problems with sleep onset.

However, current psychopathology appeared to be a major confounding factor on impaired sleep quality in our sample (attention-deficit hyperactivity disorder, depressive spectrum disorders, anxiety disorders, and substance use). This is consistent with findings from other studies investigating sleep disturbances in high-risk offspring. Psychiatric medication had no significant effect on the presence of sleep disturbances in our sample. Levenson et al. [32] reported that lifetime psychiatric history had a major effect on the presence of sleep disturbances in high-risk offspring. This limited evidence suggests that impaired sleep found in OB is secondary to psychopathology. Moreover, Jones et al. [31] did not find a significantly higher prevalence of sleep disturbances in their non-symptomatic high-risk sample.

Levenson et al. [32] found a significant circadian preference for going to bed later among high-risk offspring when compared with controls. Moreover, a circadian preference for eveningness has been found in both euthymic paediatric and adult bipolar patients [15, 40]. The trend in our high-risk sample was similar. Our finding supports the eveningness chronotype preference as a possible trait marker of BD.

Parental reports on OB using the GBISS in our study were approximately the same as the mean total scores reported by the parents of healthy children, and far lower than those reported by the parents of children diagnosed with a disorder from either the bipolar or unipolar spectrum in Meyers and Youngstrom [41]. Children themselves scored higher than parents, but according to our best knowledge, there is no published study using the self-assessment version of the GBISS. Children themselves could be more sensitive to record their sleep disturbances than their parents, but there is a lack of published GBISS data with which to compare our results. However, such discrepancy is consistent with findings from other studies examining parent versus child agreement and disagreement on presence of sleep disturbances [16, 42]. Children are more sensitive to their sleep problems than their caregivers. According to our findings, clinicians should incorporate information from both children and parents in the assessment of sleep impairment among children.

The actigraphic data showed that the OB group had more difficulties with sleep onset, and that both OB and control offspring had similar sleep macro-structure, motoric activity and circadian rhythm. On the one hand, our finding of longer sleep latency during the weekdays contrasts with Jones et al. [30], but on the other hand it is consistent with the higher occurrence of sleep-onset insomnia symptoms in our sample. The disparity between our finding and Jones et al. [30] could be caused by the difference in the current psychiatric status of the OB sample - offspring in the sample of Jones were mainly well. We did not find any rest-activity rhythm dysregulation among our OB sample. This could be explained by the timing of the actigraphic assessment. All children participating in our survey wore the actigraph during school time. Moreover, they wake up and go to bed regularly under their parents’ supervision. Future research should focus on vacation time, when children have a different daily regime than during school time.

The mismatch between subjective parental reports of sleep disturbances in offspring and objective actigraphic measures in our sample is consistent with results from other studies [43, 44]. However, the discrepancy we found in our sample may also be a matter of methodology. It has been shown that symptoms of sleep impairment are not a stable feature in children at risk of BD; rather they wax and wane episodically [44]. Therefore, a 14-day actigraphic assessment may miss this fluctuation. Parents evaluate sleep quality of their offspring in the long-term and therefore may be better able to determine sleep disturbances. Further prospective research, with a longer period of actigraphic assessment, is needed to confirm this hypothesis.

It is well known that the incidence of sleep disturbances as a comorbidity of anxiety and mood disorders is high in the child and adolescent population [45]. However, the causal relationship between sleep disturbances and anxiety and affective disorders is not well described. The question is whether altered sleep quality is a pre-existing trigger of disorder development, or whether it accompanies a psychiatric illness as a part of its manifestation. Understanding this process is important, as disturbed sleep is discussed as a possible early marker of BD [31, 46]. However, the lack of prospective sleep oriented research on offspring at high-risk for BD has prevented us from taking a final stance on this issue.

### Limitations

Several limitations related to our findings must be highlighted. First, the cross-sectional design of our study was less able to detect factors that redound to risk of illness onset in the OB. Second, the completion rate of sleep diaries was low, and consequently, we had to use the approximated bed and wake times from the PSQ in sleep macrostructure analyses. Third, the actigraphic assessment was conducted for two weeks, which might not be a sufficient period to capture impaired circadian rhythm. Fourth, the child psychiatrist who evaluated offspring was not blind to the status of the parent (control vs. bipolar). Lastly, the limited size of our sample did not allow us to separately compare symptomatic high-risk offspring to non-symptomatic high-risk offspring, as well as separate child and adolescent groups.

## Conclusions

Despite the limitations, the present findings have important implications for clinical practice. Children and adolescents at risk for BD appear to manifest symptoms of sleep disturbance, which in this study were strongly associated with current psychopathology. Clinicians taking care of anxious or depressed children and adolescents should incorporate questions aimed at sleep disturbances into their assessment, and should interview their young clients directly. Interventions aimed at both sleep impairment and psychopathology are needed for maximum treatment benefit. Further longitudinal assessment of sleep characteristics is needed to more precisely define the relationship between sleep disturbances and the development of BD.

## Abbreviations

ADHD: Attention-deficit hyperactivity disorder BD Bipolar disorder
CO: Control offspring
DSM-IV: Diagnostic and Statistical Manual of Mental Disorders, 4th Edition
GBISS: General Behavior Inventory Sleep Subscale
IS: Inter-daily stability
IV: Intra-daily variability
KSADS-PL: Kiddie Schedule for Affective Disorders and Schizophrenia Present and Lifetime version
MEQ: Morningness/Eveningness Questionnaire
NPCRA: Non-Parametric Circadian Rhythm Analysis
OB: Offspring at the genetic and environmental risk of bipolar disorder
PSQ: Pediatric Sleep Questionnaire
RA: Relative amplitude
SADS-L: Schedule for Affective Disorders and Schizophrenia Lifetime version
UK: United Kingdom
